# Interface-Based Design of High-Affinity Affibody Ligands for the Purification of RBD from Spike Proteins

**DOI:** 10.3390/molecules28176358

**Published:** 2023-08-30

**Authors:** Siyuan Song, Qinghong Shi

**Affiliations:** 1 Department of Biochemical Engineering, School of Chemical Engineering and Technology, Tianjin University, Tianjin 300350, China; 2 Key Laboratory of Systems Bioengineering and Frontiers Science Center for Synthetic Biology (Ministry of Education), Tianjin University, Tianjin 300350, China

**Keywords:** affibody, ligand design, molecular dynamics simulation, binding affinity, receptor binding domain, affinity chromatography

## Abstract

The outbreak of coronavirus disease 2019 (COVID-19) has sparked an urgent demand for advanced diagnosis and vaccination worldwide. The discovery of high-affinity ligands is of great significance for vaccine and diagnostic reagent manufacturing. Targeting the receptor binding domain (RBD) from the spike protein of severe acute respiratory syndrome-coronavirus 2, an interface at the outer surface of helices on the Z domain from protein A was introduced to construct a virtual library for the screening of Z_RBD_ affibody ligands. Molecular docking was performed using HADDOCK software, and three potential Z_RBD_ affibodies, Z_RBD_-02, Z_RBD_-04, and Z_RBD_-07, were obtained. Molecular dynamics (MD) simulation verified that the binding of Z_RBD_ affibodies to RBD was driven by electrostatic interactions. Per-residue free energy decomposition analysis further substantiated that four residues with negative-charge characteristics on helix α1 of the Z domain participated in this process. Binding affinity analysis by microscale thermophoresis showed that Z_RBD_ affibodies had high affinity for RBD binding, and the lowest dissociation constant was 36.3 nmol/L for Z_RBD_-07 among the three potential Z_RBD_ affibodies. Herein, Z_RBD_-02 and Z_RBD_-07 affibodies were selected for chromatographic verifications after being coupled to thiol-activated Sepharose 6 Fast Flow (SepFF) gel. Chromatographic experiments showed that RBD could bind on both Z_RBD_ SepFF gels and was eluted by 0.1 mol/L NaOH. Moreover, the Z_RBD_-07 SepFF gel had a higher affinity for RBD. This research provided a new idea for the design of affibody ligands and validated the potential of affibody ligands in the application of RBD purification from complex feedstock.

## 1. Introduction

Coronavirus disease 2019 (COVID-19), caused by severe acute respiratory syndrome-coronavirus 2 (SARS-CoV-2), has become a major threat to human health [1]. As of March 2023, the cumulative number of deaths was approximately 7 million [2]. Currently, it is well accepted that human angiotensin-converting enzyme 2 (hACE2) mediates viral entry by binding with the receptor binding domain (RBD) within the S1 subunit of the surface-exposed spike (S) protein on SARS-CoV-2 [3]. Within the scientific community, the S protein has not only been considered an important target for drugs and vaccine design [4], but also a promising candidate in ligand discovery used for diagnostic reagents and affinity separation [5,6,7]. For example, SARS-CoV-2 vaccination is of great importance to prevent and control COVID-19 [8]. Recently, the Coalition for Epidemic Preparedness Innovations has proposed a ‘100-day mission’ for compressing the time to launch a new vaccine to 100 days from pathogen identification [9]. However, vaccine manufacturing always includes multiple steps in the purification train (e.g., centrifugation/ultrafiltration, cell disruption, ion exchange and size exclusion chromatography, and adsorption) and suffers from long operating time, low productivity, and high manufacturing costs [10,11,12]. Therefore, the purification train is still challenging for vaccine manufacturing. Affinity chromatography is one of few techniques able to address these dilemmas [10] and has extensively been applied in bio-pharmaceutical manufacturing, especially for antibody-based products. However, there are only a few commercial paradigms of affinity chromatography in the purification of virus particles (e.g., vaccines and adeno-associated virus vectors) [10,13,14].

An important prerequisite of the application of affinity chromatography as well as diagnostic reagents is to design and develop high-affinity ligands targeted to viral particles and vaccines. Wrapp et al. found that single-domain antibodies isolated from a llama immunized with prefusion-stabilized SARS-CoV-1 S protein had cross-reactivity to SARS-CoV-2 and could neutralize SARS-CoV-2 spike pseudotyped viruses [15]. Furthermore, the release of the structure of the SARS-CoV-2 S protein [16] provides an important platform for the design and screening of affinity ligands applied in virus diagnostics and vaccine purification. As the most mature approach for ligand screening, phage display always utilizes pools of proteins/peptides randomly expressed on the phage capsid to enrich for high-affinity protein/peptide candidates for binding to the RBD of the S protein [17,18,19,20,21]. In a recent study by Yang et al. [20], five peptides were screened from a 12-mer phage display peptide library against the SARS-CoV-2 RBD. The screened peptides exhibited binding affinity to the RBD in the micromolar range and could specifically bind the inactivated SARS-CoV-2 virus. In phage display libraries, the limited library capacity makes it difficult to cover the entire sequence space theoretically accessible for random peptide libraries with more than eight variable positions [22,23]. The reduced capacity and biased population of library members become the major limitations in obtaining the best peptide desired for the targets. Compared with phage display techniques, computer-based screening could exhaust all the possibilities in a huge peptide library. In the early version of Protein Design Automation (PDA^TM^) technology, combinatorial search algorithms could search for a tractable number of sequences to satisfy the design criteria from the initial sequence space containing 10^50^ sequences or more [24]. In recent decades, computer-based screening has successfully been applied in ligand screening for protein purification [25,26,27] and therapeutic proteins with improved properties [28]. In a previous report by Chowdhury et al. [29], two promising peptides were obtained based on the rational design of peptide inhibitors targeting the spike protein of SARS-CoV-2. To date, the rational design of peptide ligands for diagnostic reagents and vaccine purification has rarely been reported.

**Figure 1:**
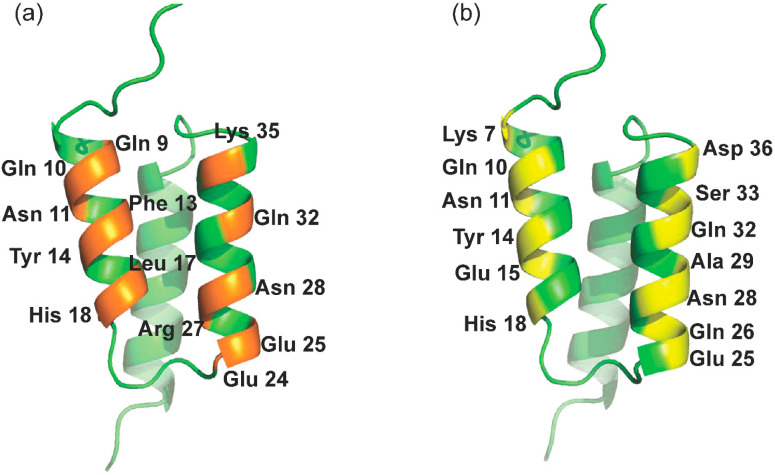
Interfaces and related solvent-accessible residues of the Z_SPA_ affibody. (**a**) Binding interfaces of the Z_SPA_ affibody; (**b**) interface for the design of RBD binding at the outer surfaces

Among all proteinaceous ligands, affibodies are an important class of protein scaffolds based on the three-helix bundle Z domain derived from *Staphylococcal* protein A (SpA) [30]. As a small and robust protein, it is capable of specific binding to different targets by the random or directed mutation of 13 solvent-accessible residues on helices α1 and α2 of the scaffold, as presented in Figure 1a [31]. In recent decades, affibodies have been extensively applied not only in affinity purification [32,33] but also in imaging [34], diagnostics, and therapeutics [35]. Malm et al. synthesized a dual-specific affibody by coupling two affibodies with albumin molecules to achieve affinity purification of HER2 and HER3 as well as prolong the in vivo half-life [33]. In August 2020, Navigo Proteins announced an artificial protein ligand for the purification of COVID-19 vaccines based on Navigo’s proprietary Precision Capturing^®^ technology [36]. However, its use in the purification of inactivated vaccines and viral-like particles was discouraged. In contrast to affinity peptide ligands, affibody ligands always have higher affinity on the order of μmol/L-pmol/L and lower nonspecific binding [28]. More importantly, protein A chromatography, as a critical and gold standard for antibody purification, provides a ready-made paradigm for the development of affibody-based chromatography to meet the mandatory requirements for clinical application, whereas this is still a challenging and laborious task for affinity peptide chromatography.

In this study, interfaces at the outer surfaces on helices α1 and α2 of the Z domain (presented in Figure 1b) were applied to construct a virtual library of Z_RBD_ affibody for the screening of high-affinity affibody ligands. Based on different strategies of affibody modeling, several Z_RBD_ affibodies with high affinity for RBD were obtained in a combination of molecular docking and molecular dynamic (MD) simulation. Then, the binding affinity of the affibody to the RBD was validated by isothermal titration calorimetry (ITC) and microscale thermophoresis (MST). Finally, the high-affinity Z_RBD_ affibodies were coupled on Sepharose 6 Fast Flow (SepFF) to evaluate the applicability of affibody chromatography in the purification of RBD.

## 2. Results and Discussion

### 2.1. Library Design and Docking to RBD

In this study, solvent-accessible residues at the outer surfaces of helices α1 (K4, K7, Q10, N11, Y14, E15, and H18) and α2 (E24, Q26, A29, Q32, S33, and D36) of the Z domain (presented in Figure 1b) were applied for the ligand design. Affibody models were constructed based on two strategies as described in the Materials and Methods section. Using the Z domain from SpA as the template [37], eight Z_RBD_ candidates were obtained (Table 1).

The affinity of Z_RBD_ to RBD was evaluated by the HADDOCK score based on molecular docking. The HADDOCK score is a weighted sum of different binding energies (van der Waals energy, electrostatic energy, desolvation energy, and so on) and other terms (e.g., buried surface areas) [38]. The docking results are listed in Table 2. In ACE2 binding with the RBD, the HADDOCK score was −117.3. It is evident from Table 2 that the binding of ACE2 and RBD was dominated by electrostatic energy. This was consistent with previously reported results [39]. Moreover, van der Waals (VDW) and desolvation energies also provided varying degrees of contribution to the docking scores [38]. In Z_RBD_ binding with RBD, all HADDOCK scores ranged from −74.1 to −137.6, but only three Z_RBD_ affibodies exhibited more favorable HADDOCK scores in RBD binding, namely, Z_RBD_-02, Z_RBD_-04, and Z_RBD_-07. In wild-type Z_RBD_ binding with the RBD, however, the HADDOCK score was only −108.6. This result further indicated that the mutation of Z_RBD_ induced a great change in electrostatic energy in complexes, and the most negative electrostatic energy was observed in the Z_RBD_-04/RBD complex, as listed in Table 2. It corresponded to the mutation of the K4 residue by glycine, a neutral residue. As the K4G mutation was substituted by the K4Q mutation (Z_RBD_-01), it led to a serious decrease in electrostatic energy in Table 2. Based on K4G mutations, the subsequent K7D mutations (Z_RBD_-05 and Z_RBD_-06) brought about a similarly great decrease in electrostatic energy. However, the dual K4Q/K7D mutation (Z_RBD_-07) led to more negative electrostatic energy than the single mutation of K4Q (Z_RBD_-01) and K4G/K7Q (Z_RBD_-05). On the other hand, Z_RBD_ binding with the RBD also exhibited more negative values of VDW energy in the complexes, and the most negative VDW energy was observed in the Z_RBD_-02/RBD complex. Therefore, Z_RBD_-02, Z_RBD_-04, and Z_RBD_-07 were further analyzed to determine the structural characteristics of the Z_RBD_–RBD complexes by MD simulation.

### 2.2. MD Simulation

#### 2.2.1. Structural Characteristics by MD Simulation

**Figure 2:**
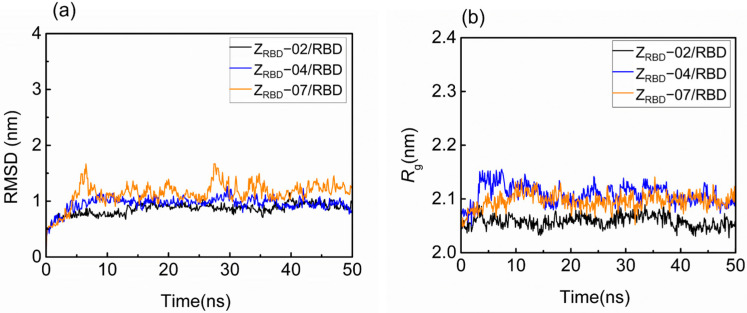
Structural fluctuation and stability of RBD–Z_RBD_ complexes in a 50 ns MD simulation. (**a**) RMSD and (**b**) *R*_g._

The structural fluctuation and stability of the complexes of the RBD and three potential Z_RBD_s were examined in a 50 ns MD simulation. The result in Figure S1 of the Supplementary Materials shows that minimal distances between the RBD and Z_RBD_ (*d*_min_) remained stable at approximately 0.11 nm for the three Z_RBD_ molecules in 50 ns, indicating that RBD and Z_RBD_ maintained effective contact [40]. Figure 2 shows the representative evolution trajectory of structural parameters, including the root-mean-square deviation (RMSD) and radius of gyration (*R*_g_). The other two sets of trajectories of structural parameters are shown in Figure S2 of the Supplementary Materials. In this study, RMSD was applied to characterize the conformational variations and atomic dynamics movements of the Cα backbone atoms of the complexes. It was observed that there was an increase in RMSD values from 0.5 to 0.8–1.5 nm in the initial 10 ns, as shown in Figure 2a. After that, all the complexes reached convergence and remained stable at 40–50 ns, indicating the limited conformational variations and good stability of the RBD complexes with three potential Z_RBD_ molecules during MD simulation. As an index of complex compactness, *R*_g_ converged rapidly into a range from 2.03 to 2.16 nm in Figure 2b, and slightly larger values of *R*_g_ were obtained in RBD binding with Z_RBD_-04 and Z_RBD_-07. Both the complexes had slightly extended structures compared with the ZRBD-02/RBD complex. In this study, Z_RBD_-02 was generated by replacing six solvent-accessible residues at the outer surface of helix α2 of the Z domain to mimic ACE2–RBD binding, as listed in Table 1. It was more favorable to form a compact complex with RBD than Z_RBD_-04 and Z_RBD_-07. In the latter, only one to two residues were substituted. The results of the MD simulation further demonstrated that the three potential Z_RBD_s obtained from molecular docking formed stable complexes with the RBD.

**Figure 3:**
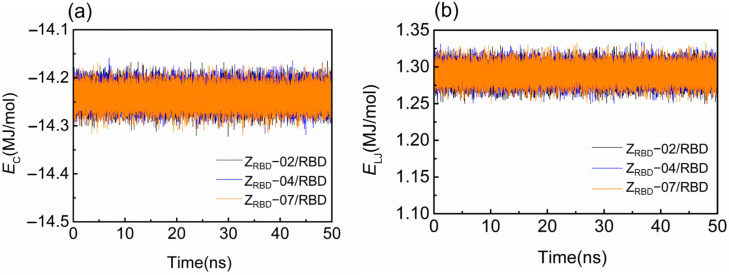
Binding energy of RBD and Z_RBD_ during MD simulation. (**a**) Coulomb energy and (**b**) L-J energy.

Figure 3 shows the representative time trajectory of the short-range Coulomb (*E*_C_) and Lennard–Jones (L-J) energies (*E*_L-J_) between the RBD and three Z_RBD_ molecules. The other two sets of time trajectories of *E*_C_ and *E*_L-J_ are shown in Figure S3 of the Supplementary Materials. In the electrically neutral environment, *E*_C_ just fluctuates around −14.1 MJ/mol during the MD simulation, as shown in Figure 3a. Such a slight fluctuation was more likely caused by the thermal motion of the complex. A similar tendency may be observed in Figure 3b, and *E*_L-J_ fluctuated at approximately 1.30 MJ/mol. Among the three complexes, negative values of *E*_C_ signified that the binding was driven by electrostatic interactions.

#### 2.2.2. Binding Free Energy Analysis

**Figure 4:**
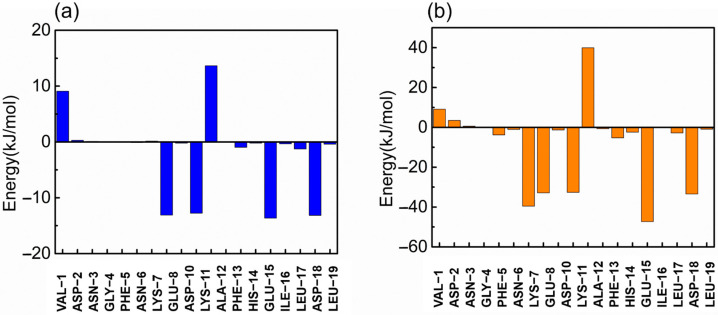
Binding free energy contribution of each residue of Z_RBD_ in the complexes. (**a**) Z_RBD_-04 and (**b**) Z_RBD_-07.

In this study, the g_mmpbsa tool of GROMACS was used to calculate the binding free energy of the RBD and Z_RBD_ by the MM-PBSA method [41]. The binding free energies (Δ*G*_bind_) of RBD complexes with Z_RBD_-02, Z_RBD_-04, and Z_RBD_-07 were determined to be −27.4, −128.6, and −405.6 kJ/mol, respectively, as listed in Table S1 of the Supplementary Materials, demonstrating that the formation of the complexes experienced a spontaneous process and that Z_RBD_-07 had the highest binding affinity to the RBD among the three potential Z_RBD_ ligands (corresponding to a more negative value in Δ*G*_bind_). In this process, electrostatic interactions (Δ*G*_elec_) dominated the formation of Z_RBD_/RBD complexes. Moreover, VDW interactions (Δ*G*_VDW_) also favored Z_RBD_ binding to the RBD whereas the polar solvation free energy (Δ*G*_PB_) disfavored binding. Herein, per-residue free energy decomposition was performed to further analyze the contribution of each residue pair to the binding free energy of the Z_RBD_-04/RBD and Z_RBD_-07/RBD complexes. The result is shown in Figure 4. In the Z_RBD_-04/RBD complex, four key residues, E8, D10, E15, and D18, of ZRBD were highlighted as shown in Figure 4a. These residues were typical of negative-charge characteristics. This result was consistent with the results of molecular docking and MD simulation described above. Compared with the Z_RBD_-04/RBD complex, moreover, an additional negatively charged residue (D7) was found in the Z_RBD_-07/RBD complex, as shown in Figure 4b. Therefore, it was confirmed that negatively charged residues played an important role in Z_RBD_ binding to the RBD.

### 2.3. Z_RBD_ Characteristics and Binding Affinity

#### 2.3.1. Spectral Characteristics of the Z_RBD_ Affibody

**Figure 5:**
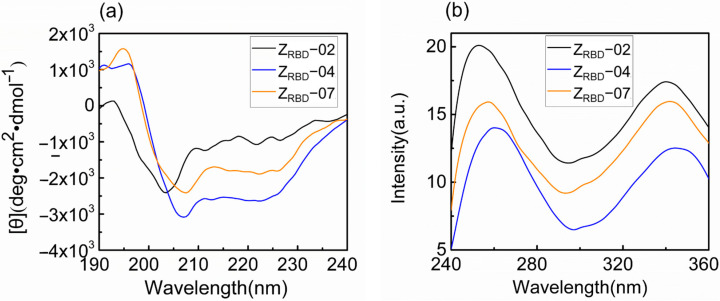
CD and FL emission spectra of Z_RBD_ affibodies. (**a**) CD spectra, (**b**) FL emission spectra.

In this study, the spectral characteristics of the Z_RBD_ affibody were measured with circular dichroism (CD) and fluorescence (FL) spectrometers. As shown in Figure 5a, the CD spectra exhibited dual negative peaks at 208 and 222 nm and a positive peak at 195 nm. This was consistent with the characteristics of the CD spectrum of the Z domain from SpA reported previously [42]. Among the three CD spectra, these characteristic peaks were more pronounced in the Z_RBD_-07 affibody. The FL emission spectrum was applied to analyze the tertiary structure of Z_RBD_ in solution. The results in Figure 5b show that Z_RBD_ had a maximum emission intensity at approximately 340 nm, indicating a stable tertiary structure of the affibody in solution. However, a slight red shift of the FL emission peak was observed in Z_RBD_-04, demonstrating that the chromogenic Tyr residue in the native state was exposed to a slightly more hydrophilic environment and led to a slight change in tertiary structure [43]. Spectral results confirmed that the three Z_RBD_s maintained their molecular structure and that Z_RBD_-02 and Z_RBD_-07 were more stable in solution.

#### 2.3.2. Binding Affinity of the Z_RBD_ Affibody to the RBD

**Figure 6:**
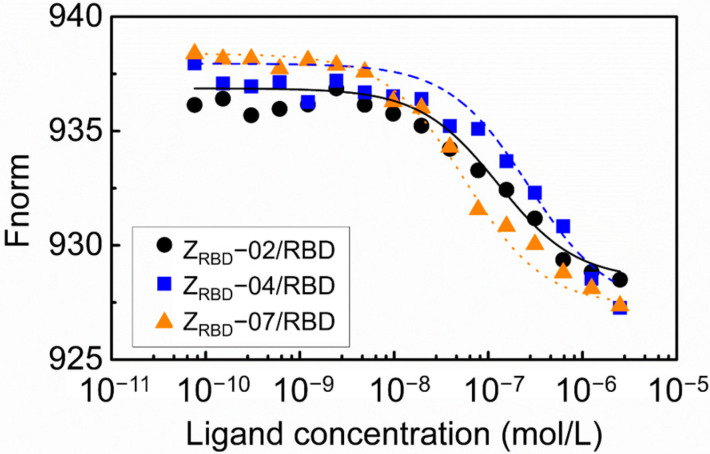
Concentration–response curve of the binding interaction between the Z_RBD_ and RBD by MST.

In this study, the binding affinity of affibody to the RBD was evaluated with microscale thermophoresis (MST). The results in Figure 6 show that the binding of Z_RBD_ and RBD led to a marked variation in normalized fluorescence, and all the curves had a reverse sigmoidal shape in the thermophoresis signal with an increase in affibody concentration until the binding approached saturation. By fitting the normalized fluorescence signal and affibody concentration, the dissociation constants of RBD binding were determined to be 133.4 nmol/L for Z_RBD_-02, 377.3 nmol/L for Z_RBD_-04, and 36.3 nmol/L for Z_RBD_-07. These values of the dissociation constant were superior to those in the peptide–RBD binding (80–970 nmol/L) [44]. The results indicated that Z_RBD_-02 and Z_RBD_-07 had higher affinity for the RBD than Z_RBD_-04 and even the binding affinity of Z_RBD_-07 was an order of magnitude higher than that of Z_RBD_-04. In this study, the raw ITC data in Figure S4 of the Supplementary Materials further showed that the binding of Z_RBD_-02 and Z_RBD_-07 exhibited typical exothermic characteristics. This evidence was consistent with previous results of MD simulations.

### 2.4. Chromatographic Performance

**Figure 7:**
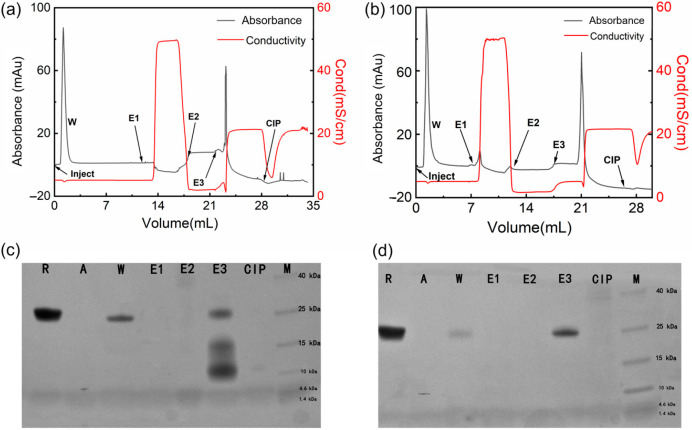
Binding and elution of the RBD by affibody-based chromatography. (**a**) Chromatographic result with Z_RBD_-02 SepFF; (**b**) Chromatographic result with Z_RBD_-07 SepFF; (**c**) SDS–PAGE image of the purification of the RBD by Z_RBD_-02 SepFF; (**d**) SDS–PAGE image of the purification of the RBD by Z_RBD_-07 SepFF. In the SDS–PAGE images: R, raw material; A, affibody ligand; W, washing fraction; E1, elution fraction by 0.5 mol/L NaCl; E2, elution fraction by 0.1 mol/L Gly-HCl buffer; E3, elution fraction by 0.1 mol/L NaOH; CIP, 0.5 mol/L NaOH; M, protein marker.

In this study, Z_RBD_-02 and Z_RBD_-07 with the C-terminal CK tag were coupled to thiol-activated SepFF gel to synthesize two affinity adsorbents (Z_RBD_-02 SepFF and Z_RBD_-07 SepFF) for chromatographic experiments. The ligand densities were determined to be 6.5 mg/mL gel for Z_RBD_-02 SepFF and 7.3 mg/mL gel for Z_RBD_-07 SepFF. The chromatographic results are shown in Figure 7. In the chromatographic process, the bound component was eluted with 0.5 mol/L NaCl (pH 7.5, E1), 0.1 mol/L Gly-HCl buffer (pH 3.0, E2), and 0.1 mol/L NaOH (pH 13, E3). The collected fraction was analyzed by SDS–PAGE. As shown in Figure 7a,b, the bound component was just eluted by 0.1 mol/L NaOH. The SDS–PAGE images in Figure 7c,d likely indicate that there was no band observed in lanes E1 and E2. Previously, Dutta et al. reported that, at acidic pH 3.5, RBD could effectively elute from an NGL COVID-19 affinity adsorbent, in which an artificial protein–ligand was coupled to Praesto^®^ Epoxy 85 resin [45]. A harsher elution in this study manifested stronger RBD binding on Z_RBD_-02 SepFF and Z_RBD_-07 SepFF gels. The SDS–PAGE results further showed that lane E3 of Figure 7d had a darker band than the corresponding lane in Figure 7c at the same sample loading. This result indicated that the Z_RBD_-07 SepFF gel had a higher binding amount of RBD at a similar ligand density. Moreover, low-molecular-weight components in lane E3 of Figure 7c may be dimeric Z_RBD_-02 via disulfide linkage because the matrix-assisted laser desorption ionization time-of-flight (MALDI-TOF) mass spectrum (Figure S5 of the Supplementary Materials) showed a characteristic peak at 6790 *m*/*z*, very similar to the characteristic peak of Z_RBD_-02 in MS analysis. Corresponding to RBD elution, it may also be observed in Figure 7a,b that the unbound component was washed from the chromatographic column after sample injection. Based on the SDS–PAGE images in Figure 7c,d, it could be affirmed that the washing fraction included RBD due to the same mobility between lanes R and W. More importantly, more RBD was washed from the column packed with Z_RBD_-02 SepFF, as shown in Figure 7c. It was corroborative that Z_RBD_-07 had a higher affinity than Z_RBD_-02. This conclusion was consistent with the MST evidence in Figure 6. The binding and elution results of RBD demonstrated that Z_RBD_ affibodies, especially Z_RBD_-07, were great potential ligands in the purification of RBD and medical diagnosis of COVID-19.

## 3. Materials and Methods

### 3.1. Materials and Chemicals

SepFF gel was purchased from Cytiva (Uppsala, Sweden). Z_RBD_ affibodies and affibodies with a C-terminal CK tag (95% purity) were synthesized by Ziyu Biotech Co., Ltd. (Shanghai, China). Recombinant His-tagged SARS-CoV-2 Spike RBD (amino acids Arg319-Phe541-His6) and hACE2 expressed in SF9 insect cells were obtained from Nankai University [46]. HPLC-grade acetonitrile was obtained from Merck KGaA (Darmstadt, Germany). Trifluoroacetic acid (TFA) was purchased from Alfa Aesar (Heysham, UK). Dimethyl sulfoxide (DMSO), epichlorohydrin (ECH), 2,2′-dithiodipyridine (DPDS), dithiothreitol (DTT), and sodium thiosulfate were received from Heowns Biochem Technologies (Tianjin, China). Unless otherwise specified, other chemical reagents used in this work were purchased from local suppliers.

### 3.2. Affibody Modeling

In this study, solvent-accessible residues at the outer surfaces of helices α1 and α2 of the Z domain (presented in Figure 1b) were applied for ligand design. The candidate affibodies were generated based on two strategies. In strategy I, K4 and K7 on helix α1 of the Z domain were replaced by negatively charged/polar residues (Q and D) and neutral residues (G) to investigate the charge influence of residues in RBD binding. In strategy II, solvent-accessible residues at the outer surfaces of helices α1 and α2 were replaced by key residues on ACE2 involved in RBD binding as reported by Nord et al. [47]. All the Z_RBD_ affibodies in the library were built manually via homology modeling. The sequence of the recombinant Z domain from SpA (PDB code: 2SPZ) was retrieved in FASTA format from the Protein Data Bank for the preparation of amino acid sequences of the Z_RBD_ affibody in this study. The modeling of the affibodies’ structures was performed by SWISS-MODEL (Biozentrum, Switzerland). Once designed, either Z_RBD_ affibodies or Z_RBD_ affibodies with a C-terminal CK tag (Z_RBD_-Cys-Lys) were synthesized.

### 3.3. Docking of Z_RBD_ Affibody to the RBD of Spike Protein

In this research, a highly fuzzy-driven docking method named HADDOCK (high ambiguity driven protein–protein docking) was used to create the model of the RBD–affibody complex. HADDOCK permits the utilization of biochemical or biophysical interaction data, such as chemical shift perturbation data or mutation data from NMR titration experiments, and information about interaction residues is introduced as ambiguous interaction constraints (AIRS) to drive the docking process [48]. Crystal structures of the RBD of the SARS-CoV-2 spike protein in complex with ACE2 (PDB ID: 6M0J) and ACE2 (PDB ID: 1R42) were acquired from the Protein Data Bank (http://www.rcsb.org/pdb/, accessed on 30 March 2022). Prior to docking, the RBD and Z_RBD_ affibodies were prepared by PyMOL software to remove other protein chains, water molecules, and ions from the PDB file and insert missing H-atoms. After two prepared PDB files were uploaded, the amino acid 470–510 region was selected as the RBD docking region and the amino acid 1–19 region was selected as the affinity ligand docking region according to the binding mechanism of RBD to ACE2 [21,49]. The docking protocol requires free protein and fuzzy-interaction-constrained PDB files, including orientation randomization and rigid body energy minimization, semirigid simulated annealing in torsional space, and explicit solvent refinement of Cartesian space. After calculation, the structures were ranked according to the weighted sum of various energy terms (electrostatic, VDW, desolvation, buried surface area, etc.) [38]. The final structure was aggregated by using pair–backbone RMSD at the interface and analyzed according to the average interaction energies (*E*_elec_, *E*_vdw_, *E*_AIR_, and *E*_desolv_).

### 3.4. Molecular Dynamics Simulation

The structures of SARS-CoV-2 RBD and affibody complexes used for model construction were built from HADDOCK. The CHARMM36 force field was used and the TIP3P model was used for water molecules. In this study, three simulation systems were constructed using models including RBD complexes with different Z_RBD_ affibodies. In simulation system 1, a regular dodecahedral box was generated to solvate the RBD and Z_RBD_-02, which contains 10,536 water molecules and 1 chloride ion (3 more sodium ions were used as counter ions to keep the system electrically neutral). In simulation system 2, a regular dodecahedral box was generated to solvate the RBD and Z_RBD_-04, which contains 10,441 water molecules and 1 chloride ion (3 more sodium ions were used as counter ions to keep the system electrically neutral). In simulation system 3, a regular dodecahedral box was generated to solvate the RBD and Z_RBD_-07, which contains 10,463 water molecules and 1 chloride ion (4 more sodium ions were used as counter ions to keep the system electrically neutral).

MD simulations in the NVT ensemble were performed using GROMACS version 2018.1-gpu (http://www.gromacs.org/, accessed on 10 October 2022). The temperature was controlled at 300 K with a time constant of 0.1 ps using the velocity-rescale (v-rescale) method [50]. The cutoffs of the neighboring atom list, L-J potential, and Coulomb potential energies were all set to 1.2 nm. The initial velocity of the particles was generated according to the Maxwell distribution at 300 K. The coordinates were saved every 2 ps. The periodic boundary condition was used in the *x*, *y*, and *z* directions. After the energy was minimized using the steepest descent method, a 50 ns MD simulation was performed as described. Every MD simulation was repeated to obtain three independent simulation trajectories for sufficient sampling and analysis.

In this study, RMSD and *R*_g_ as well as *d*_min_ were calculated to quantitatively evaluate the stability of protein constructs. RMSD was calculated by the rms program of GROMACS software. The larger the RMSD, the larger the structural change during docking. *R*_g_ was calculated by the gyrate program of GROMACS software. The smaller the *R*_g_, the more compact the molecular structure. The g_mmpbsa tool from GROMACS was applied in the implementation of the molecular mechanics Poisson–Boltzmann surface area (MM-PBSA) approach for end-state free energy calculations from MD trajectories and binding free energy decomposition [41]. Therefore, L-J and Coulomb energies were calculated using the g_mmpbsa tool in GROMACS software to describe the interaction between the RBD and Z_RBD_ affibodies.

### 3.5. Spectral Characterization of Z_RBD_

CD spectra of free affibodies were determined with a JASCO J-810 spectro-polarimeter from JASCO Inc. (Easton, MD, USA) with a 1 mm path cuvette at room temperature. In this study, 20 mmol/L PB buffer (pH 7.5) was used as the sample buffer unless otherwise specified. Prior to the analysis, the affibodies were diluted to 0.2 mg/mL with the sample buffer. The data were collected at 0.1 nm intervals at a scanning speed of 100 nm/min in a wavelength range of 190–240 nm. In the measurement, the sample buffer was used as a reference. After three consecutive wavelength scans were taken for each sample, the spectra were averaged and then corrected by subtracting the spectrum of a reference.

FL emission spectra of free affibodies were analyzed with a PerkinElmer LS-55 fluorescence spectrophotometer in the range of 280–400 nm (emission slit width of 7.5 nm) at the excitation wavelength of 284 nm (excitation slit width of 12.0 nm). All the samples were measured in a quartz colorimetric dish with a 10 mm path, and the scanning speed was 300 nm/min. The spectral analysis was repeated three times for each sample. The fluorescence background was determined using the sample buffer, and the emission spectra of the samples were corrected by subtracting the FL background.

### 3.6. Binding Affinity Experiments

Heat variation in the affibody and RBD binding was measured with an Affinity ITC from TA Instrument Company (New Castle, DE, USA) at 25 °C and a stirring speed of 200 rpm. Prior to the ITC experiment, affibody samples were dissolved in the sample buffer with a final concentration of 250 μmol/L, and the RBD stock solution was diluted to 22 μmol/L with the sample buffer. After the samples were degassed at 25 °C for 10 min, the degassed RBD solution (500 μL) was slowly transferred into the titration cell, and the affibody solution was inhaled into the ITC syringe. In the titration, the affibody solution was injected into 21 injections of 2 μL each at intervals of 300 s. As a reference, the sample buffer was injected into the RBD solution to determine the heat of dilution. All experiments were repeated in triplicate.

MST of Z_RBD_ binding to RBD was performed with a Monolith NT.115 Microscale Thermophoresis from Nano Temper Technologies GmbH (Munich, Germany). Prior to the measurement, the dye was labeled on the RBD via a C-terminal His_6_ tag using a Monolith His-Tag Labeling Kit. The concentration of the labeled RBD was 0.05 μmol/L, and the concentrations of the Z_RBD_ affibodies were in a gradient dilution from 2.5 μmol/L in 16 PCR tubes. After the labeled RBD was transferred into PCR tubes, the reaction was incubated and then loaded into Monolith NT.115 capillaries. MST was carried out in MST buffer (20 mmol/L PB buffer, 0.1% Tween-20, pH 7.5) at 20% red channel LED and 20% MST power.

### 3.7. Synthesis of Affibody-Based Gels and Chromatographic Performance

**Figure 8:**
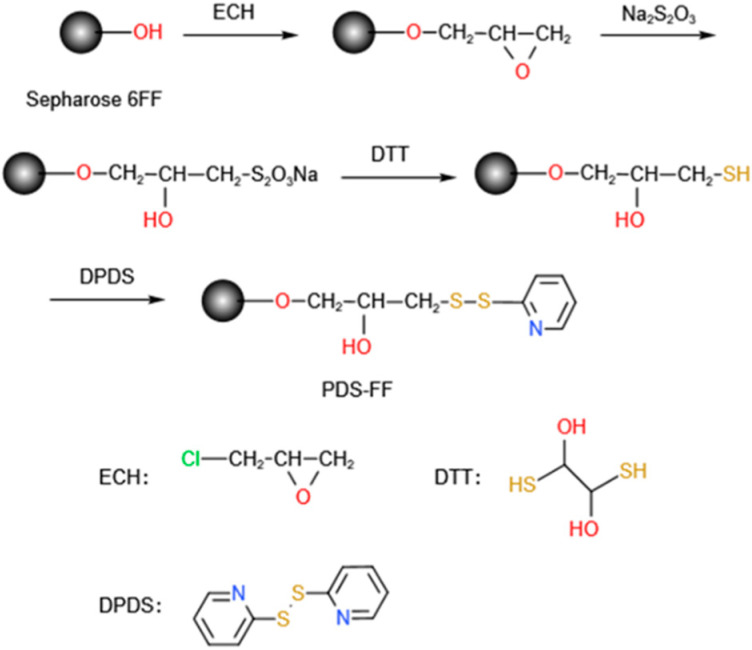
Synthesis of SepFF-MPy gel.

Z_RBD_ affibodies with a C-terminal CK tag were coupled onto SepFF via the thiol immobilization technique. It included the synthesis of a thiol-modified gel (presented in Figure 8) followed by ligand coupling. In this study, the reaction was carried out in a water bath at 170 rpm unless otherwise specified. In brief, SepFF was first activated by reacting with ECH under alkaline conditions, as described previously [51]. Then, the activated SepFF (3 g) was mixed with 2 mol/L sodium thiosulfate (3 mL), and the mixture was reacted at 25 °C for 6 h. The resulting gel was rinsed with excess water and resuspended in 0.2 mol/L NaHCO_3_ (3 mL). After 0.17 g/L DTT in 1 mmol/L EDTA solution (3 mL) was added, the mixture was reacted at 25 °C for 30 min. The gel was rinsed with 0.2 mol/L NaHCO_3_ and 1 mmol/L EDTA solution, and dried in a G3 sintered glass funnel. Mercaptopyridine (MPy) was coupled to the gel as described by Ferraz et al. [52]. After the drained gel was washed with an acetone–water mixture (3/2 *v*/*v*) containing 0.06 mmol/L EDTA and 20 mmol/L NaHCO_3_ (50 mL), the drained gels (3.0 g) were mixed with an acetone–water mixture (3/2 *v*/*v*, 5 mL) and 0.3 mol/L DPDS (10 mL). The slurry was reacted at 25 °C for 1 h. The product was collected and washed with an acetone–water mixture (3/2 *v*/*v*) and 1 mmol/L EDTA and denoted SepFF-MPy.

In the coupling of Z_RBD_ affibodies, Z_RBD_-02 and Z_RBD_-07, onto SepFF-M, 20 mmol/L PB buffer (pH 7.5) was used as the coupling buffer. After 1.0 g SepFF-MPy was washed with the coupling buffer, the gels were mixed with 10 mg Z_RBD_ affibody in coupling buffer (5 mL), and the slurry was reacted at 25 °C for 1.5 h. Then, cysteine was added, and residual MPy on the gels was replaced by cysteine after reacting continuously for 30 min. In this study, two final products were denoted Z_RBD_-02 SepFF and Z_RBD_-07 SepFF for chromatographic experiments.

Chromatographic experiments were performed at 0.2 mL/min (1.0 cm/min) using a Tricorn 5/50 column packed with Z_RBD_ SepFF gel (1 mL) connected to an AKTA Purifier 10 chromatography system (GE Healthcare, Uppsala, Sweden). After the column was equilibrated with adsorption buffer (20 mmol/L PB buffer, pH 7.5), the RBD sample was injected, and then the column was washed with 6 column volumes (CVs) of adsorption buffer to remove unbound components. Finally, the column was eluted with 0.5 mol/L NaCl in adsorption buffer, 0.1 mol/L Gly-HCl buffer (pH 3.0), and 0.1 mol/L NaOH. The collected eluted fraction at pH 3.0 was neutralized with 1.0 mol/L Tris-HCl buffer (pH 8.0) at a proportion of 5% (*v*/*v*). Cleaning in place (CIP) was carried out with 0.5 mol/L NaOH. The purity of the RBD in the flow-through and elution fractions was analyzed by SDS–PAGE and the leakage of affibody ligand was determined with a MALDI-TOF mass spectrometer.

### 3.8. Sample Analysis

The change in affibody content during ligand coupling was measured with a Shimadzu Essentia LC-16 chromatographic system (Kyoto, Japan) using an Ultimate^®^ LP-C18 column from Welch Materials Inc. (West Haven, CT, USA) at 220 nm using 0.2% TFA in water as mobile phase A and 0.2% TFA in acetonitrile as mobile phase B. In the analysis, a linear gradient of mobile phase B from 20% to 70% in 25 min was applied. Purity analysis was performed with 15% non-reducing SDS–PAGE gel. The electrophoresis was run at a constant voltage of 120 V until the dye front reached ~1 cm from the bottom of the gel. The gel was stained with Coomassie brilliant blue R-250. After decolorization, the gel was analyzed by imaging and grayscale calculation using the gel analysis system from Shenhua Technology to determine the purity of the RBD. The elution components were analyzed with a Bruker Autoflex III MALDI-TOF mass spectrometer (Bruker Daltonics, Leipzig, Germany). The spectrum from 5 kDa to 8 kDa was collected in positive ion mode.

## 4. Conclusions

In this study, the interface at the outer surface of helices α1 and α2 on the Z domain from SpA was introduced for ligand design. Based on different strategies for affibody modeling, eight Z_RBD_ candidates were generated and three Z_RBD_ affibodies with lower HADDOCK scores than the ACE2/RBD complex, Z_RBD_-02, Z_RBD_-04, and Z_RBD_-07, were obtained. MD simulation verified that the three Z_RBD_ affibodies formed stable complexes with the RBD and this process was driven by electrostatic interactions. Based on the per-residue free energy decomposition, four residues with negative-charge characteristics were further highlighted for the formation of the complexes. CD and FL emission spectra showed that the Z_RBD_ affibodies maintained their molecular structure in solution and this phenomenon was more pronounced for Z_RBD_-02 and Z_RBD_-07. Although three Z_RBD_ affibodies had a binding affinity to RBD, higher binding affinities were obtained in the binding of the RBD with Z_RBD_-07 (*K*_d_ = 36.3 nmol/L) and Z_RBD_-02 (*K*_d_ = 133.4 nmol/L). Herein Z_RBD_-02 and Z_RBD_-07 affibodies were selected for chromatographic verifications. Chromatographic results showed that both Z_RBD_-02 SepFF and Z_RBD_-07 SepFF gels could bind the RBD effectively and the latter had a higher binding amount of RBD. The bound RBD was eluted by 0.1 mol/L NaOH, indicating strong binding of the RBD on Z_RBD_ SepFF gels. This research provided a new idea for the design of affibody ligands, and the results demonstrated that affibodies derived from the Z domain of SpA were a potential protein scaffold for the design of high-affinity ligands in the application of RBD purification from complex feedstock.

## Figures and Tables

**Table 1:** Affibody library for ligand screening.

No.	Affibody Modules
Z_RBD_-01	K4Q
Z_RBD_-02	K4Q, E24Q, Q26T, A29D, F30K, Q32H, S33E
Z_RBD_-03	K4Q, K7H, Q10T, N11Y, Y14Q, E15D, I16K
Z_RBD_-04	K4G
Z_RBD_-05	K4G, K7D
Z_RBD_-06	K4G, K7D, H18F
Z_RBD_-07	K4Q, K7D
Z_RBD_-08	K4Q, Q10E

**Table 2:** HADDOCK scoring and energic contribution of complexes of Z_RBD_ and RBD.

Complex	HADDOCK Score	Van der Waals Energy (kJ/mol)	Electrostatic Energy (kJ/mol)	Desolvation Energy (kJ/mol)	Restraints Violation Energy (kJ/mol)	Buried Surface Area (Å^2^)
Z_RBD_/RBD	−108.6 ± 2.6–97.8 ± 2.4	−66.8 ± 7.0	−204.4 ± 43.9	−14.3 ± 4.3	45.1 ± 38.6	1741.9 ± 49.9
Z_RBD_-01/RBD	−97.8 ± 2.4	−51.3 ± 5.2	−161.9 ± 30.6	−17.7 ± 1.5	36.1 ± 20.6	1567.6 ± 36.2
Z_RBD_-02/RBD	−137.6 ± 5.2	−80.6 ± 8.2	−243.4 ± 27.8	−23.7 ± 1.4	153.8 ± 14.2	2094.8 ± 76.4
Z_RBD_-03/RBD	−74.1 ± 5.8	−75.9 ± 2.1	−276.2 ± 11.3	−22.2 ± 4.0	79.24 ± 9.15	2093.6 ± 49.0
Z_RBD_-04/RBD	−132.7 ± 1.9	−71.6 ± 7.0	−290.0 ± 25.8	−19.1 ± 1.5	160.6 ± 17.6	2137.7 ± 40.7
Z_RBD_-05/RBD	−117.7 ± 3.4	−60.9 ± 5.8	−187.2 ± 41.8	−22.9 ± 3.0	34.3 ± 20.9	1679.5 ± 37.3
Z_RBD_-06/RBD	−114.1 ± 3.5	−65.1 ± 3.6	−109.8 ± 3.9	−31.1 ± 2.8	41.2 ± 32.0	1609.3 ± 50.8
Z_RBD_-07/RBD	−125.3 ± 7.5	−65.6 ± 6.7	−219.8 ± 11.1	−20.1 ± 1.3	43.9 ± 12.4	1756.0 ± 91.0
Z_RBD_-08/RBD	−109.5 ± 13.2	−57.2 ± 9.3	−184.8 ± 55.9	−21.4 ± 6.9	61.0 ± 36.8	1805.6 ± 152.2
ACE2/RBD	−117.3 ± 3.6	−52.6 ± 4.0	−261.2 ± 42.2	−15.9 ± 5.1	34.1 ± 20.8	1840.3 ± 39.6

## Data Availability

Data will be made available on request.

## Supplementary Materials

The following supporting information can be downloaded at: https://www.mdpi.com/article/10.3390/molecules28176358/s1, Figure S1: Minimal distances between RBD and Z_RBD_ affibodies; Figure S2: Structural fluctuation and stability of RBD–Z_RBD_ complexes in a 50 ns MD simulation. Two sets of simulation data of (a) RMSD and (b) *R*_g_; Figure S3: Binding energy of RBD and ZRBD during MD simulation. Two sets of simulation data of (a) Coulomb energy and (b) L-J energy; Figure S4: Calorimetric titration of RBD with Z_RBD_ affibodies at 25 °C: (a) titration of Z_RBD_-02 to RBD; (b) titration of Z_RBD_-07 to RBD; Figure S5: MALDI-TOF mass spectrum of eluted fraction by 0.1 mol/L NaOH in a Z_RBD_-02 SepFF column; Table S1: Binding free energies of Z_RBD_/RBD complexes.

## Sample Availability

Samples of the compounds are available from the authors.
