# Supplementary material for: Interface-Based Design of High-Affinity Affibody Ligands for the Purification of RBD from Spike Proteins
Source: Molecules. 2023 Aug 30;28(17):6358. doi: 10.3390/molecules28176358 (PMC10489752; doi:10.3390/molecules28176358)
Supplement: Supplementary file 1 [file molecules-28-06358-s001.zip › molecules-2549403-supplementary.docx]

Supplementary material

Interface-based design of high-affinity affibody ligands for the purification of RBD from spike proteins

Siyuan Song ^1^, and Qinghong Shi ^1,2,^*

^1^ Department of Biochemical Engineering, School of Chemical Engineering and Technology, Tianjin University, Tianjin 300350, China

^2^ Key Laboratory of Systems Bioengineering and Frontiers Science Center for Synthetic Biology (MOE), Tianjin University, Tianjin 300350, China

***** Correspondence: qhshi@tju.edu.cn





**Figure S1** Minimal distances between RBD and Z_RBD_ affibodies


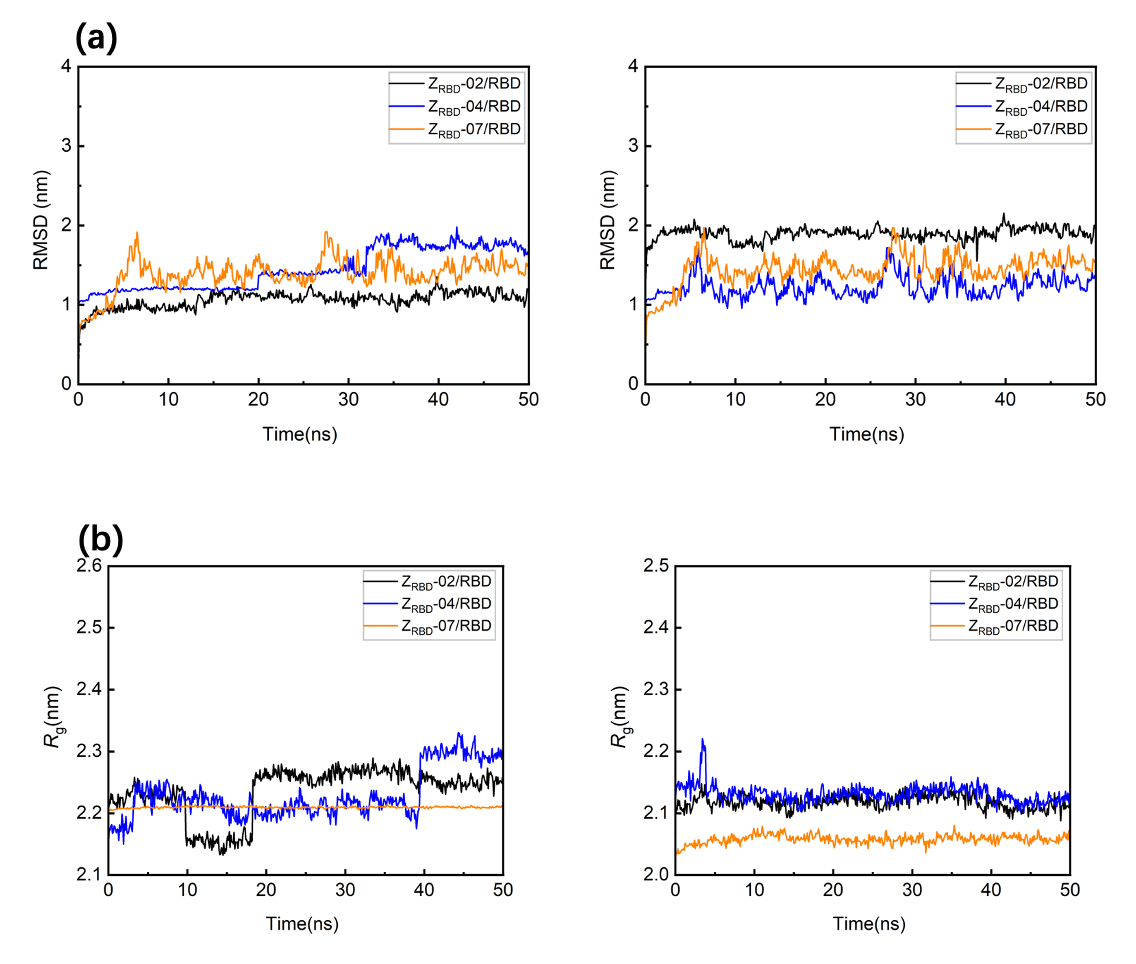


**Figure S2** Structural fluctuation and stability of RBD-Z_RBD_ complexes in a 50-ns MD simulation. Two sets of simulation data of (a) RMSD and (b) R_g_


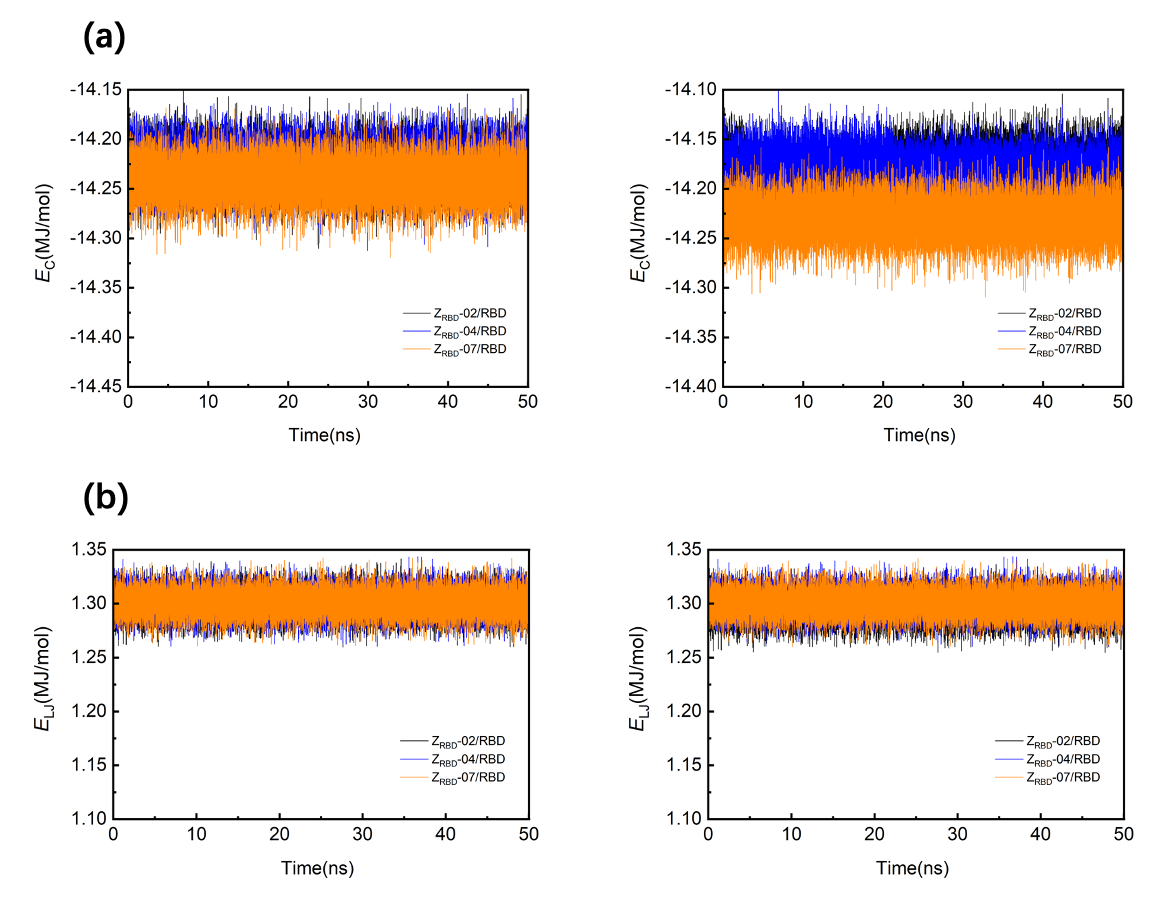


**Figure S3** Binding energy of RBD and Z_RBD_ during MD simulation. Two sets of simulation data of (a) Coulomb energy and (b) L-J energy.


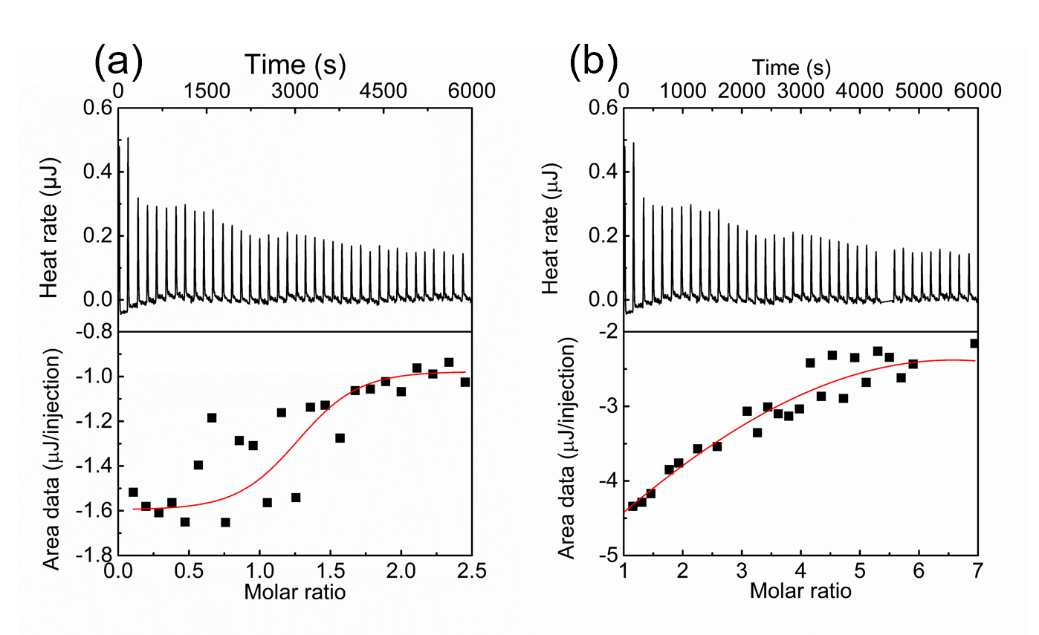


**Figure S4** Calorimetric titration of RBD with Z_RBD_ affibodies at 25°C

(a) Titration of Z_RBD_-02 to RBD; (b) Titration of Z_RBD_-07 to RBD





**Figure S5** MALDI-TOF mass spectrum of eluted fraction by 0.1 mol/L NaOH in Z_RBD_-02 SepFF column

**Table S1** Binding free energies of Z_RBD_/RBD complexes

| **Complexes** | **Δ*G*_vdw_**  **(kJ/mol)** | **Δ*G*_SASA_**  **(kJ/mol)** | **Δ*G*_elec_**  **(kJ/mol)** | **Δ*G*_PB_**  **(kJ/mol)** | **Δ*G*_bind_**  **(kJ/mol)** |
| --- | --- | --- | --- | --- | --- |
| Z_RBD_-02/RBD | -0.029±0.002 | -0.134±0.198 | -86.3±0.7 | 59.5±7.1 | -27.4±7.2 |
| Z_RBD_-04/RBD | -44.4±8.7 | -6.631±1.304 | -178.4±19.7 | 101.4±19.6 | -128.6±13.6 |
| Z_RBD_-07/RBD | -200.1±4.9 | -26.46±0.7 | -637.2±11.4 | 458.2±11.3 | -405.6±10.4 |
